# COVID-19: A Psychosocial Perspective

**DOI:** 10.3389/fpsyg.2020.554624

**Published:** 2020-12-01

**Authors:** Syed Hassan Raza, Wajiha Haq, Muhammad Sajjad

**Affiliations:** ^1^School of Economics, Quaid-i-Azam University, Islamabad, Pakistan; ^2^Department of Economics, School of Social Sciences and Humanities, National University of Sciences and Technology, Islamabad, Pakistan; ^3^School of Energy and Environment, City University of Hong Kong, Kowloon, Hong Kong

**Keywords:** public health, psychology, stress, COVID-19, well-being

## Abstract

The World Health Organization declares coronavirus disease 2019 (COVID-19) as a pandemic, and The World Economic Forum argues that the COVID-19-induced global lockdown is the biggest psychological experiment. This study is an attempt to empirically evaluate the possible adverse psychosocial effects caused by COVID-19-related lockdown, if any. To do so, a cross-sectional study is conducted based on a comprehensive online survey using snowball sampling to analyze the level of social and psychological impacts (i.e., stress, belief in stakeholders, fear of losing job, and life satisfaction) during the early stage of the outbreak in Pakistan. The questionnaire is filled out by the residents in Pakistan including working professionals and students (sample size is 428). We find that the development of stress due to COVID-19-induced lockdown is particularly because of mood swings. Additionally, a higher prevalence of stress in the children of highly educated mothers is evident (95% confidence). To assess the belief in stakeholders, we focus gender, demographics, and education. It is observed that parental education and age significantly affect the belief in several stakeholders (i.e., government, media, religious clerics, and family). The lockdown-induced fear of losing job is lower in female and male children whose fathers are graduates. Lastly, we observe that food storage and “no fear of losing job” significantly increases the odds of life satisfaction. These findings have important implications in the context of social insurance, parental education, and policy related to COVID-19 at various levels. This study further facilitates to understand the factors that might affect the mental health and life satisfaction of people during such pandemics.

## Introduction

The world is facing one of the most dangerous challenges in our lifetime due to the outbreak of the novel coronavirus (COVID-19), which has now spread to almost every country (211 countries and territories to be specific) on the global map (Pettersson et al., 2020). The effects caused by COVID-19 apart from adverse health are becoming eminent in different dimensions such as social, psychological, and economic. COVID-19 affected the first patient in late November 2019, and the World Health Organization (WHO) declared the phenomena as a Public Health Emergency of (international concern) in January 2020 (World Health Organization [WHO], 2020). COVID-19, also known as severe acute respiratory syndrome coronavirus 2 (SARS-CoV-2), is a major outbreak after SARS-CoV-1, which spread in the year 2002. As of 18th April 2020, about 1.5 million people have been confirmed to be affected by COVID-19 and more than 1,00,000 are dead due to this global outbreak. As an optimal solution, WHO has advised a quarantine policy to limit the spread of the virus, and now more than one-third of the global population is under some form of isolation (Kaplan et al., 2020).

COVID-19 outbreak has changed the current living arrangement via social isolation, fear of human-to-human transmission, and closure of educational as well as business institutes. This situation could very well lead to severe psychological impacts on societies (Fardin, 2020), which is well evident as the consequences of previous outbreaks such as the recent Ebola (Van Bortel et al., 2016) and Middle East respiratory syndrome coronavirus (MERS-CoV) (Al-Rabiaah et al., 2020). The outbreak of Ebola in Guinea, Liberia, and Sierra Leone adversely affected the quality of life and led to social, psychological, and economic breakdown (Van Bortel et al., 2016). Fear and anxiety in the patients of COVID-19 have already been observed (Xu et al., 2020). Belief in governments during the flu pandemic has been found significantly associated with gender, whereas income, age, education, and people living in urban and rural areas were found to have no effect on belief in the government that they will do actions to benefit the community. Gender, education, and income were found to be correlates of belief in the family (Paek et al., 2008). Life satisfaction during COVID-19 was also tested during pandemic (Satici et al., 2020; Zacher and Rudolph, 2020). Another study investigated that age, income, and gender are significant correlates of life satisfaction during COVID-19 in Germany. However, the effect of education on life satisfaction during COVID-19 pandemic was found insignificant (Zacher and Rudolph, 2020). Similarly, the existing literature in the field of epidemiology has well recognized the impact of diseases on the behavior and psychology of individuals (Cherif et al., 2016; Pellmar et al., 2020).

Humans, known as social animals, living in social clusters might suffer not only from COVID-19 itself but through the isolation as well. Theorists define the perceived social isolation as physical separation from others and tested this phenomenon for having any psychological consequences. They found that social isolation can result in negative emotions (e.g., anger, sadness, and low mood), decreased levels of arousal in extroverts, and those who prefer to stay around individuals and even decline in cognitive abilities (e.g., problem solving and decision making; Cacioppo and Hawkley, 2009). Consequently, overwhelming feelings of isolation or the feeling of loss of social relations have been shown to have implications for the decline in trust in relations and belief, alongside a buildup of worsening immune functioning, disruptions in sleep, and laziness, which lead to weight gain and stress (Cacioppo et al., 2002). These theories have also been used with regard to studying how social disconnection can lead to the emergence of stress, fear, suicidal ideation, and risks of early mortality (Holt-Lunstad et al., 2010). Therefore, the need to have social connection is a core human characteristic and, if violated, can bring many consequences including mood swings, stress, fear, and decline in belief and trust on family or other relations. Human beings cannot remain isolated for longer periods, as it results in the development of fear, stress, distrust, depression, and associated negative feelings. The outbreaks are known for disturbing societal psychology (Van Bortel et al., 2016; Lee et al., 2018; Al-Rabiaah et al., 2020; Fardin, 2020). Wang et al. (2020) evaluated the psychological responses to analyze the impact of COVID-19 on psychology. However, the evidence on the psychological impacts of COVID-19 and the consequent social-distancing-induced isolation is scarce, as it has roughly been 4 months since the pandemic started around December in China. The need for studying the psychological outcome of COVID-19 is pivotal particularly in low-income countries where the Happiness Index is substantially low and people are struggling to improve their living standards (United Nations, 2020). This study will evaluate the psychological impacts (positive/negative) of COVID-19 that can eventually help in making corrective decisions and resource allocation to support societal life satisfaction taking the public perception into account.

In connection to this, we assess the psychological influences of COVID-19-induced lockdown, if any, from different perspectives in Pakistan, a developing country in Asia. Pakistan is geographically located between two epicenters of the COVID-19 outbreak—China and Iran (Saqlain et al., 2020)—making it suitable to study the psychological impacts of this current outbreak. This study is an initial effort to document the varied psychological effects in connection to COVID-19-related social-distancing and lockdown situation. As of April 18th, 2020, Pakistan has 7,516 confirmed cases of COVID-19, and the country is practicing a lockdown situation from the last 4 weeks including the closure of public/private business centers and educational institutions along with a very limited market activity. Pakistan first took measures on February 28, 2020 by closing the borders with Iran followed by China suspecting departure of affected people. Schools were closed nationwide on March 1st, 2020. After 2 months of complete lockdown, the country shifted to a partial lockdown.

The main aim of this study is to stimulate theoretical perspectives and novel investigations on how the COVID-19-induced quarantine is psychologically affecting the public. To do so, we first identify the presence of stress and fear in the respondents due to COVID-19 and then evaluate different associated factors such as the belief of individuals in major stakeholders such as family, government, media, and religious clerics toward COVID-19 response and the education of respondents and their parents. The COVID-19 pandemic did not only spread disease but was also supplemented with the lockdown as well. As the observed lockdown is first of its nature in the current century and the disease is novel, it is important to analyze the psychological effects on people. The study is novel due to nature of the problem and is one of the initial attempts, particularly in Pakistan. The study has analyzed different demographic and sociological factors affecting stress, trust, and belief of people during the lockdown. The fear of job loss and life satisfaction was also analyzed during lockdown. The findings from this study would progressively further the understanding regarding the psychological impacts of such pandemics providing policy-related implications, which can ensure effective public health-relevant management. Hence, this study has not only practical implications for current times but will also advance the theory for further research.

## Materials and Methods

**FIGURE 1:**
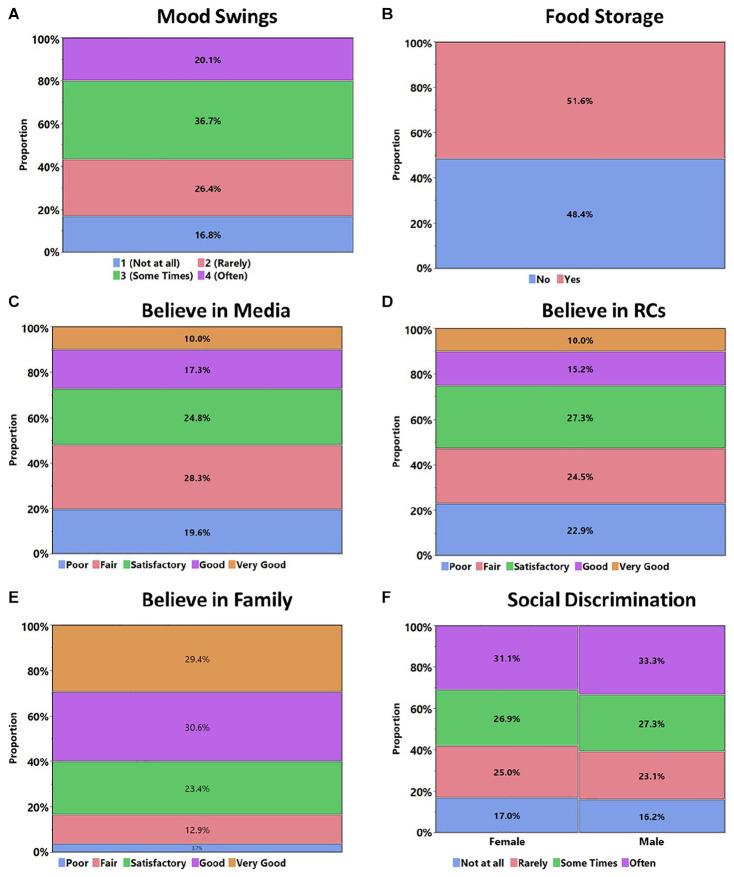
Frequency distribution of key indicators of respondents.

The overall workflow of the study consists of five main steps: questionnaire design → pilot survey → revising the questionnaire for the nature of questions and clarity → final survey → impact analysis. To begin with, previous surveys are comprehensively reviewed (Leung et al., 2003; Leung et al., 2009; Rubin et al., 2010; Wang et al., 2020), and the most relevant questions are selected to design the final questionnaire. Questions on demographic, socioeconomic, and psychological aspects during the COVID-19 outbreak are the key constituents of the questionnaire. For example, respondents are asked about their gender, age, education, occupation, living arrangement, household size, and the education of their parents. Additionally, we emphasize asking about fear, stress, mood swings, laziness, belief, and trust in different associated actors (i.e., government, family, and media). The variables are operationally defined and asked particularly in the context of COVID-19-induced lockdown. The variable of “fear” includes fear of losing job due to lockdown, fear of being diagnosed with COVID-19, and fear that a family member will be diagnosed with COVID-19 leading to some social stigmatization. In order to assess the “belief” in social and government institutions, respondents are asked how well they believe that their family, government (i.e., federal, provincial, and local), medical services providers, and religious clerics responded to COVID-19 pandemic. It is noted that the questions are asked for each category separately. The “trust” variable is measured by how well the respondent trusts the organizations in the context of COVID-19 pandemic response. The Likert scale is used to measure the fear, belief, trust, stress, mood swings, and laziness as a consequence of COVID-19-induced lockdown. Belief was measured on Likert scale of 5 ranging from “poor” to “very good.” The five-category Likert scale of life satisfaction ranges from “very unsatisfied” to “very satisfied.” We present the distribution of mood of swings in Figure 1A, food storage in Figure 1B, believe in media in Figure 1C, believe in religious clerics in Figure 1D, believe in family in Figure 1E, and social discrimination in Figure 1F. Stress was measured using the 4-point Likert scale ranging from “not at all” to “often” based on the Depression Anxiety Stress Scale (DASS) (Lovibond and Lovibond, 1995).

Similar to many other countries, the government of Pakistan has restricted gatherings, and staying at home is advised to reduce the human interactions during this pandemic. The country is experiencing a lockdown situation from the last 4 weeks. Therefore, the respondents are also asked whether they have stored food due to lockdown or not. In addition, respondents are asked whether they think the relief package of PKR 12,000 by the federal government is sufficient or not. It is noted that the responses to these questions are dichotomous.

We use the questionnaire-based cross-sectional survey across Pakistan to evaluate the psychological response during this pandemic of COVID-19, if any. Assuring the safety of respondents and keeping the paradigm in mind, the best possible way in this current situation is the online surveying approach to collect the data (Wang et al., 2020). Using a snowball sampling strategy, an anonymous online questionnaire is floated among individuals. It is initially floated among the residents of Islamabad Capital Territory (ICT), Pakistan, including working professionals and students. They were further encouraged to pass it on to others. In addition to this, we approach people residing in all the provinces in Pakistan, electronically, and ask them to float the questionnaire further so that we can cover a wider area. The data are collected during the last week of March and first week of April 2020, and we receive 560 responses in total. After data cleaning for incomplete responses and randomly selecting a balanced mix of male and female respondents, we are left with 428 responses, which are used in further analysis. Simple random sample may lead to a sample that does not truly reflect the population makeup. The sample may over- or underrepresent a demographic such as gender. Stratified techniques are used to overcome this problem. According to this technique, pools according to categories are formed from which the subsample is randomly selected to better represent the population (Pérez Salamero González et al., 2016). It is important to note that our sample of 428 observations is enough for generalization with a 5% margin of error at 95% confidence as per the sample size calculation criteria discussed in Krejcie and Morgan (1970). Hence, we are confident to say that the survey data are stable, and it is appropriate to use the data for further analyses procedures (DeVellis, 1991; Wu et al., 2020).

To analyze the association between different COVID-19-related outcomes (i.e., stress, trust, belief, fear of job loss, and life satisfaction) with potential explanatory factors (Table 1), we use the multivariate logistic regression because the dependent variables in our analysis are categorical. The general equation is as follows:

**TABLE 1:** Detail on the dependent and independent variables used in this study to set different logistic models.

**Dependent variables → independent variables ↓**	**Stress due to lockdown**	**Belief in family’s response**	**Belief in media’s response**	**Belief in religious clerics’ response**	**Fear of losing job**	**Life satisfaction**
Gender	**✓**	**✓**	**✓**	**✓**	**✓**	**✓**
Age	**✓**	**✓**	**✓**	**✓**	**✓**	**X**
Marital status	**✓**	**✓**	**✓**	**✓**	**✓**	**X**
Household size	**✓**	**✓**	**✓**	**✓**	**✓**	**X**
Profession	**✓**	**✓**	**✓**	**✓**	**X**	**X**
Living arrangement	**✓**	**✓**	**✓**	**✓**	**✓**	**X**
Trust in provincial gov	**X**	**X**	**X**	**X**	**X**	**✓**
Trust in family	**✓**	**X**	**X**	**X**	**X**	**✓**
Relief package	**X**	**X**	**X**	**X**	**X**	**✓**
Mood swings	**✓**	**X**	**X**	**X**	**X**	**X**
Fear of losing job	**✓**	**X**	**X**	**X**	**X**	**✓**
Life satisfaction	**✓**	**X**	**X**	**X**	**X**	**X**
Food storage	**X**	**X**	**X**	**X**	**X**	**✓**
Respondent’s education	**✓**	**✓**	**✓**	**✓**	**✓**	**X**
Mother’s education	**✓**	**✓**	**✓**	**✓**	**✓**	**X**
Father’s education	**✓**	**✓**	**✓**	**✓**	**✓**	**X**
						

$$\theta \left(Y=k|X=x_{m\,i}\right)=logit\delta \left(x\right)=\ln\left[\frac{\delta \,\left(x\right)}{1-\delta \,\left(x\right)}\right]=\beta_{o\,k}+\beta_{1\,k}x_{1\,i}+\beta_{2\,k}x_{2\,i}+\beta_{3\,k}x_{3\,i}+\ldots +\beta_{n\,k}x_{n\,i}$$

where “Y” is a vector for dependant variable having k outcomes, and “X” is a vector for independent variables. The number of observations is given by “*i*,” and “m” denotes the number of independent variables. The detail on independent and dependent variables is provided in Table 1. We have only reported significant variables in the results from the models, whereas, for the rest of the variables in the model, please refer to Table 1. The Benjamini–Hochberg procedure (Thissen et al., 2002) has been used to get the adjusted *p*-values to reduce the chance of false positive results (type 1 error). The omitted variable test has also been used to check the omitted variable bias and model misspecification following Papke and Wooldridge (1996). The insignificant *p*-value shows that the model is correctly specified. In addition, while using the logistic regression, the test of parallel line assumptions has also been carried out. The significant test shows that the assumption of parallel lines for the usage of ordinal analysis has been violated. In addition, the model fit has further been carried out. In this study, we use JMP Pro. Software, a comprehensive suite of computer programs for statistical analysis from the SAS Institute, United States of America^1^.

## Results

**FIGURE 2:**
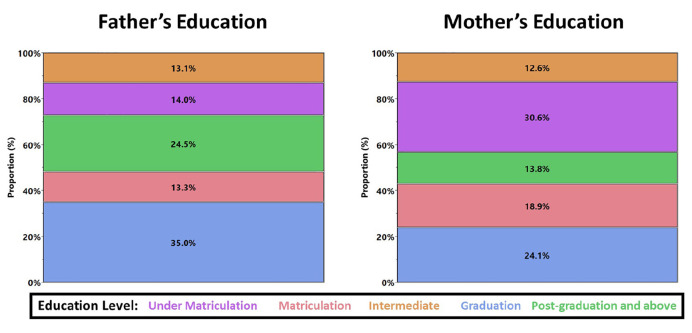
Frequency distribution of parental education of respondents.

Pakistan has a relatively young population, which is also reflected from the responses as the mean age of the respondents is 25 years ranging between 18 and 55 years. The descriptive analysis shows that around 77% of the respondents are single and the rest are married. In the context of the working population, we observe that 32% of the respondents are currently working and 48% hold a graduation degree in the sample (*n* = 428). The overall respondents are widespread across the country belonging to 50 distinct districts. Most of the respondents (89.5%) are living with family, which might be due to strong family bonding and the concept of social insurance in Pakistan. Besides, we observe that ∼55% of respondents are having a household size of three to six people, and ∼35% of respondents have more than six people, reflecting a relatively higher household size in Pakistan. In general, the education of respondents’ fathers is higher than their mothers (Figure 2). For example, the proportion of “undermatriculation”—the lowest level of education in the survey—for mothers is ∼31% as compared to 14% for fathers. Similarly, the proportion of “postgraduation and above” for fathers is higher than that for mothers (∼25 and ∼14%, respectively). Therefore, it becomes interesting to explore the role of parents’ education in developing stress-related outcomes, especially when people are spending a lot of time with their families under the lockdown situation. From the life satisfaction perspective, it is even more compelling to document the role of parental education in “overall life satisfaction.” In short, we compile a diverse group of respondents of different ages, living in distinct areas, with different household size, and having different family backgrounds.

### Socioeconomic Factors

It is observed that ∼51% of the respondents have stored food during this lockdown (Figure 1B). Most of those respondents have a family size of four to six people (56%) and more than six household sizes (45%). This seems legitimate that respondents with larger household sizes tend to store food during emergencies such as COVID-19, as the duration of the current lockdown is not clear at the moment. More than 50% of the respondents said that quarantine, staying at home, and social distancing have affected their lives to some context. In addition, ∼70% think that working from home has not increased their workload. Respondents are also asked which challenge Pakistan is facing currently. Around 61% of respondents think that COVID-19 is the biggest challenge right now. On the other hand, 32% consider the economic crisis as the biggest challenge. Respondents were also asked about the existence of pandemic response policy. Around 37% of people think that Pakistan has a pandemic response policy, which, in fact, does not exist. This represents a false public perception toward government measures to deal with current and future pandemics such as COVID-19, which could potentially lead to ineffective/non-serious precautions to tackle the pandemics. Among those who think that Pakistan has a pandemic response policy, ∼50% are graduates. The proportion of the respondents who think the response policy exists is larger for female (41%) than for male respondents (33%). Similarly, a reasonable proportion of the respondents (∼32%) think that if they tested positive for COVID-19, they will face social discrimination often. Among these respondents, the proportion of male is slightly larger than the female respondents, ∼33% as compared to ∼31%, respectively (Figure 1F). A major reason behind this larger male proportion might be that men in Pakistan have more leverage to go outside as compared to women.

### COVID-19-Related Stress

**FIGURE 3:**
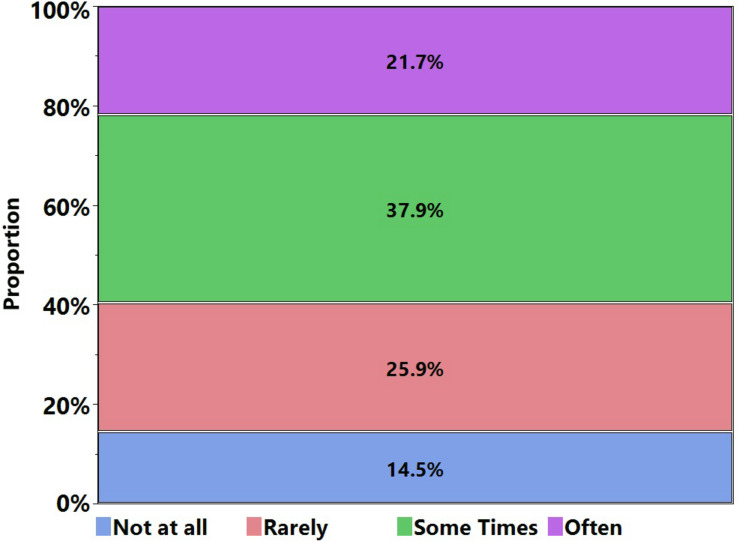
Stress distribution of respondents.

Stress is the most evident outcome of any outbreak (Al-Rabiaah et al., 2020; Wang et al., 2020). Stylized facts from this study reveal that approximately 38% of the respondents are stressed “sometimes,” and 22% of them are stressed “often” in the context of lockdown related to COVID-19 (Figure 3). As mentioned earlier, Pakistan is under lockdown situation from the last 4 weeks with a clear government advisory to stay at home and avoid all sorts of gatherings. In this regard, we evaluate the association of stress with the age of the respondent, parental education, household size, and mood swings. The results show that stress is being affected by age, parent’s education, and mood swings due to the lockdown (Table 2). It is noted that the results are only presented for the statistically significant factors at a 95% confidence level. If the respondent is not having mood swings at all, he/she has a higher probability that he/she might not experience stress at all. Therefore, a demonstration of mood swings might have become a source of elaboration of stress, and similarly, having mood swings “often” increases the odds of experiencing stress “often.” Likewise, experiencing mood swings “rarely” increases the odds of having stress “rarely.” It is evident from the results that if the mother has secondary school certification (matriculation) as compared to intermediate, then they have higher odds of having “no stress due to lockdown at all.” But if the mothers of respondents are “postgraduate or above” as compared to “intermediate,” then they have lower odds of having stress “rarely.” This situation indicates that the respondents having highly educated mothers have higher odds of having stress “often.” Highly educated mothers can sense the severity of the situation and unintentionally transfer it to children, which might result in higher stress. Furthermore, if the overall life satisfaction is “good” and “satisfactory,” the respondents have higher odds of relatively less stress (i.e., odds of having stress “rarely” and “sometimes” are higher as compared to “often” simultaneously). This result is self-explanatory that if life satisfaction is high, then the probability of having stress “often” is low.

**TABLE 2:** Multilogistic-regression-based results for stress.

**Dependent variables**	**Independent variables**	**Odds ratio (Std. Errors)**	***p*-value**	**Adjusted *p*-value**	**Chi square**
**Stress due to COVID-19-related lockdown (base category “often”)**					
	**Stress due to lockdown (not at all)**				
	Mother’s education matriculation	3.001 (0.412)	0.007*	0.030	7.12
	Mother’s education postgraduate or above	0.212 (0.601)	0.010*	0.030	6.61
	Mood swings not at all	4.314 (0.376)	0.0001*	0.010	15.05
	**Stress due to lockdown (rarely)**				
	Mother’s education postgraduate or above	0.413 (0.420)	0.035*	0.042	4.42
	Mood swings rarely	1.944 (0.292)	0.022*	0.037	5.18
	Life satisfaction level good	1.879 (0.311)	0.042*	0.042	4.12
	**Stress due to lockdown (sometimes)**				
	Age	1.089 (0.041)	0.040*	0.042	4.20
	Father’s education matriculation	0.408 (0.368)	0.015*	0.030	5.89
	Working status (students)	1.883 (0.284)	0.026*	0.037	4.95
	Life satisfaction level satisfactory	1.885 (0.257)	0.013*	0.030	6.08
Unadjusted R^2^			0.164		
**Effect likelihood ratio tests**					
Independent variables			Prob. > chi-square		
Mood swings			< 0.0001*		
Life satisfaction			0.013*		
Education			0.010*		
**Whole model test**					
Difference (−loglikelihood)			< 0.0001*		
**Omitted variable test**					
Model			0.7328		
**Test of parallel lines**					
Difference (−2loglikelihood)			< 0.0001*		

*^∗^*p* < 0.05.*

### Belief in Response to COVID-19

#### Belief in Family

Belief is measured on a scale of “poor,” “fair,” “satisfactory,” “good,” and “very good.” The results show that the respondent’s age along with parent’s education are significant factors to affect the respondents belief in response to COVID-19 outbreak by his/her family (Table 3). We further find that increasing age or elderly people have lesser odds of having “poor” belief in the family’s response to COVID-19 as compared to “very good.” If the respondent’s fathers have “postgraduation or above” education as compared to intermediate, they have lower odds of having “fair” belief in the family as compared to “very good.” On the contrary, if mothers have “postgraduation or above” education as compared to intermediate, they have lower odds of having “satisfactory” belief in the family as compared to “very good.” In short, the respondents with highly educated parents have a strong belief that their family has responded well to COVID-19 outbreak. Conclusively, elderly people and respondents whose parents have “postgraduation or above” have a higher belief in the family’s response to COVID-19.

**TABLE 3:** Multilogistic-regression-based results for belief in family’s response to COVID-19.

**Dependent variables**	**Independent variables**	**Odds ratio (Std. Errors)**	***p*-value**	**Adjusted *p*-value**	**Chi square**
**Belief in family’s response to COVID-19 (base category “very good”)**					
	**Family’s response (Poor)**				
	Age	0.762 (0.136)	0.046*	0.046	3.970
	**Family’s response (fair)**				
	Father’s education Post Graduate or Above	0.242 (0.540)	0.008*	0.028	6.870
	**Family’s response (satisfactory)**				
	Mother’s education Post Graduate or above	0.339 (0.441)	0.014*	0.028	6.000
	Mother’s education matriculation	1.900 (0.308)	0.037*	0.046	4.340
Unadjusted R^2^			0.133		
**Effect likelihood ratio tests**					
Independent Variables			Prob. > Chi square		
Marital status			0.044*		
Household Size			0.002*		
Mother’s education			0.003*		
**Whole model test**					
Difference (−loglikelihood)			< 0.0001*		
**Omitted variable test**					
Model			0.1033		
**Test of parallel lines**					
Difference (−2loglikelihood)			< 0.0010*		

*^∗^*p* < 0.05.*

#### Belief in Media

The odds of female respondents are lower for having “poor” belief as compared to “very good.” It implies that female respondents have a higher belief in the media’s response as compared to male respondents (Table 4). This brings an interesting insight regarding the female individual’s reliability over media for the response to COVID-19. Thereafter, we document that elderly people also have lower belief in the media’s “poor” response in the context of COVID-19 as compared to “very good.” This implies that they are satisfied with the media’s reporting and role in making people aware of COVID-19 situations, or in other words, they believe that media somehow fulfilled their responsibility toward COVID-19 communication. If the mother’s education is “under matriculation” (secondary school certification), the odds are higher for having “poor” belief in media. The same trend follows for “fair” belief. Whereas, if the mother’s education is “postgraduation or above” as compared to “intermediate,” then the odds of having “fair” belief as compared to “good” are lower. The respondents having highly educated mothers have a higher probability of having a “very good” belief in the media’s response to this current pandemic. Therefore, we observe the acceptance of media with higher attainment of mothers’ education. Additionally, the media’s role is questionable as well; one can argue the inability of media to form a better opinion among the mothers with lower educational attainment.

**TABLE 4:** Multilogistic-regression-based results for belief in media’s response to COVID-19.

**Dependent variables**	**Independent variables**	**Odds ratio (Std. Errors)**	***p*-value**	**Adjusted *p*-value**	**Chi square**
**Belief in media’s response to COVID-19 (base category “very good”)**					
	**Media’s response (poor)**				
	Gender (female)	0.576 (0.218)	0.011*	0.020	6.350
	Age	0.902 (0.052)	0.048*	0.048	3.880
	Mother’s education under matriculation	3.343 (0.482)	0.012*	0.020	6.260
	**Media’s response (fair)**				
	Mother’s education postgraduate or above	0.348 (0.491)	0.032*	0.048	4.600
	Mother’s education under matriculation	3.842 (0.465)	0.003*	0.015	8.360
Un-adjusted R-square			0.088		
**Effect likelihood ratio tests**					
Independent Variables			Prob. > Chi square		
Gender			0.014*		
**Whole model test**					
Difference (−loglikelihood)			0.0388*		
**Omitted variable test**					
Model			0.5408		
**Test of parallel lines**					
Difference (−2loglikelihood)			< 0.0001*		

*^∗^*p* < 0.05.*

#### Belief in Religious Clerics

Our results show that as one grows older, the odds increase for having “fair” and “satisfactory” belief in religious clerics as compared to odds of “very good” (Table 5). Similarly, the respondents whose mother’s education is “postgraduation or above” have higher odds of having “fair” and “good” belief than “very good.” Thus, the belief in religious clerics for their response to the pandemic is significantly influenced by the age of the respondents and their mother’s education (95% confidence). Respondents having highly educated mothers and elderly have lesser belief in religious clerics. This is interesting to observe that the odds of having higher belief in religious clerics are linked with mother’s education instead of that with the father in Pakistan. This is due to the fact that mothers usually spend more time with the family, and educated mothers are more likely to educate their children at an early age about religion and importance of different stakeholders. On the other hand, majority of the fathers remain engaged in the labor market or daily life routines out of their homes. Therefore, they usually have less time to spend with the family to discuss the importance or the role of religious clerics during such pandemics. In the past, Pakistan has experienced SARS, MERS, H1N1 flu, and dengue (Khan et al., 2008; Hakim et al., 2011; Aamir et al., 2012; Saqib et al., 2017). In the history of Pakistan, religious clerics have not been active in case of any endemic and have not played a pivotal role in spreading awareness about the outbreaks of different diseases or taking any necessary measure. This might have led to a situation where the educated group has lesser belief in religious clerics.

**TABLE 5:** Multilogistic-regression-based results for belief in religious clerics’ response to COVID-19.

**Dependent variables**	**Independent variables**	**Odds ratio (Std. Errors)**	***p*-value**	**Adjusted *p*-value**	**Chi square**
**Belief in religious cleric’s response to COVID-19 (base category “very good”)**					
	**Religious clerics response (fair)**				
	Age	1.159 (0.067)	0.026*	0.035	4.92
	Mother’s education postgraduate or above	3.919 (0.689)	0.047*	0.047	3.93
	**Religious clerics response (satisfactory)**				
	Age	1.166 (0.065)	0.018*	0.035	5.57
	**Religious clerics response (good)**				
	Mother’s education postgraduate or above	5.512 (0.707)	0.015*	0.035	5.83
Unadjusted R^2^			0.097		
**Effect likelihood ratio tests**					
Independent variables			Prob. > Chi square		
Gender			0.011*		
Age			0.047*		
**Whole model test**					
Difference (−loglikelihood)			0.0064*		
**Omitted variable test**					
Model			0.7306		
***Test of parallel lines***					
Difference (−2loglikelihood)			0.0370*		

*^∗^*p* < 0.05.*

### Fear of Losing Job in Response to COVID-19

The results show that the working female respondents (81%) have no fear of losing job. Among those who are working, the respondents with higher education [i.e., graduates (38%) and postgraduates (50%)] have no fear of losing job. Since our reference category in this case is “Yes,” the respondents whose father’s education is “matriculation” as compared to “intermediate” have lower odds of “no of fear of losing job” (Table 6). In contrary, we observe that the respondents whose father’s education is “graduation” as compared to “intermediate” have higher odds of “no fear of losing job.” This signifies that the role of a father’s education cannot be undermined in terms of psychological consequences during such pandemics. Our findings ascertained that people with lower educational attainment are more susceptible to this economic insecurity as a consequence of COVID-19 that they will be laid off, whereas we do not observe such estimates for women or people with higher educational attainment. It is noted that the model used for fear is simple logistic instead of the multivariate logistic model due to the nature of responses.

**TABLE 6:** Logistic-regression-based results for fear of losing job due to COVID-19-induced lockdown/quarantine.

**Dependent variables**	**Independent variables**	**Odds ratio (Std. Errors)**	***p*-value**	**Adjusted *p*-value**	**Chi square**
**Fear of losing job due to COVID-19-related lockdown (base category “yes”)**					
	**Fear of losing job**				
	Gender (female)	1.427 (0.138)	0.010*	0.019	6.61
	Father’s education graduation	1.762 (0.242)	0.019*	0.019	5.49
	Father’s education under matriculation	0.501 (0.280)	0.013*	0.019	6.08
Unadjusted R^2^			0.134		
**Effect likelihood ratio tests**					
Independent variables			Prob. > Chi square		
Gender			0.009*		
Father’s education			0.020*		
**Whole model test**					
Difference (−loglikelihood)			< 0.0001*		
**Omitted variable test**					
Model			0.3688		

*^∗^*p* < 0.05.*

### COVID-19 and the Public Perception Toward the Life Satisfaction

Human life satisfaction is a broad concept representing how well people meet their emotional, environmental, spiritual, social, physical, and economic needs. It also includes individuals’ own judgment about their own life and society (Levy and Guttman, 1975; Levy and Sabbagh, 2008; Jowell and Eva, 2009). The results from the descriptive analysis show that around 38% of the respondents remain neutral about their life satisfaction. Around 57% of male and 43% of female respondents are satisfied with their life during this pandemic. Among those people who have responded “very satisfied” even in this pandemic, 37% have postgraduate education. Thus, the perception about the life satisfaction of female and highly educated individuals even in this pandemic seems not to be much influenced. Higher educational attainment is linked with higher premium or security for long-term success in the labor market. This might be the potential reason that a larger proportion of people with higher education is satisfied even during this emergency.

Table 7 shows that female, as compared to male, respondents have higher odds of having “not satisfied life” in this pandemic as compared to “very satisfied life.” If people have no fear of losing job, the odds of “not at all satisfied” are lower as compared to “very satisfied.” “No fear of losing job” has lower odds of being “not at all satisfied” and “little satisfied” as compared to “very satisfied” with life. In Pakistan, the job market is not precipitating except for low-/daily wage workers. That is why having fear of losing job has not influenced the perception of individuals about life satisfaction. Additionally, the respondents have higher odds of “satisfied” as compared to “very satisfied” when they have no food storage. In Pakistan, people are hoarding food, and thus, having food storage might increase the satisfaction level and assure life sustainability.

**TABLE 7:** Multilogistic-regression-based results for public perception toward life satisfaction in the face of COVID-19.

**Dependent variables**	**Independent variables**	**Odds ratio (Std. Errors)**	***p*-value**	**Adjusted *p*-value**	**Chi square**
**Current life satisfaction (base category “very satisfied”)**					
	**Life satisfaction (not at all)**				
	Gender (female)	1.786 (0.276)	0.036*	0.036	4.39
	Fear of losing job (No)	0.535 (0.297)	0.035*	0.036	4.42
	**Life satisfaction (little satisfied)**				
	Fear of losing job (no)	0.889 (0.204)	0.002*	0.008	8.85
	**Life satisfaction (satisfied)**				
	Food storage (No)	1.437 (0.155)	0.019*	0.036	5.45
Unadjusted R^2^			0.074		
**Effects likelihood ratio tests**					
Independent variables			Prob. > Chi square		
Fear of losing job			0.001*		
Food storage			0.032*		
**Whole model test**					
Difference (−loglikelihood)			0.0002*		
**Omitted variable test**					
Model			0.1010		
**Test of parallel lines**					
Difference (−2loglikelihood)			0.0090*		

*^∗^*p* < 0.05.*

## Conclusion

In connection to WHO’s considerations about the psychological impacts of COVID-19 pandemic, this study is considered an initial effort to provide a thorough evaluation of the possible impacts of COVID-19-induced lockdown on public psychology—in Pakistan. For this purpose, an online survey is conducted, with a 5% margin of error (95% confidence), and several statistical approaches are employed to analyze different factors and to establish on how people might have been psychologically affected by the current COVID-19-induced isolation. Based on the initial results presented here, it can be argued that the outbreak has posttraumatic effects on public psychology. The results show that parental education is a significant factor associated with the stress level among the respondents during this quarantine situation. Respondents of older age and having parents with higher education have a higher belief in their family during COVID-19 outbreak. People have a lesser belief in “response by religious clerics.” Highly educated people have a high probability of having no fear of losing job. Women have a higher probability of less satisfaction with their life during this pandemic. This outbreak has affected the life satisfaction of society and caused stress.

This study indicates that the role of parental education during such pandemics cannot be undermined especially the role of father’s education. Therefore, the policy-making institutions must focus on spending more on the current generations’ education to cope with such challenges in the future. This will further remove the economic insecurity in the people of Pakistan, as we document higher odds in fear of losing jobs among less-educated people. Moreover, higher education, less fear of losing jobs, and no stress will contribute positively toward the life satisfaction in the people, which is ideal not only to have a happy population but also to have highly productive labor. Lastly, Pakistan is a religious country where religious clerics are the stakeholder in every major decision making. Thus, the government must mobilize all the resources to convince religious clerics to take up the responsibility and contribute more in terms of educating people regarding such psychological consequences in the wake of pandemics such as COVID-19.

The governments need to focus on the mental health of societies, as they might get more stressed with prolonged lockdown situation, carrying minimal out-of-house activity, and having “fear of losing job” (e.g., among the less educated such as daily wagers). To cope with this situation, counselors and psychologists can also play an important role through providing their voluntary services for the life satisfaction of society during this pressing time of global emergency. The study is important in the context of psychological interventions to improve future pandemic response in a more resilient way.

The authors do acknowledge the intrinsic limitations of the current study at this point. First of all, due to the lockdown situation, we had to rely on the online surveying approach, which might have some issues (i.e., inability to reach challenging population such as elderly population and people with no educational background). However, online surveys are a robust and cost-effective means to systematically gather the data from a wider range of audiences. It not only increases the response rate but also saves time, which is a critical aspect of the current situation. A further in-person survey or mix-method approach can be used to survey the same questions once the lockdown is over to check the sensitivity of the online survey, if any. Furthermore, we do not, particularly, emphasize the socioeconomic conditions of the respondents, which could help in evaluating the correlation of outcomes to tailor necessary interventions. Lastly, although the global time period for COVID-19 is roughly 4 months, the pandemic surged in Pakistan in March 2020. This implies that the study is performed in an early outbreak situation and is only focused on Pakistan. Follow-up large-scale studies could progressively help to assess progression of psychological manifestations. However, this might have to wait until the imminent threat of COVID-19 subsides.

## Data Availability Statement

The raw data supporting the conclusions of this article will be made available by the authors, without undue reservation, to any qualified researcher.

## Ethics Statement

Ethical review and approval was not required for the study on human participants in accordance with the local legislation and institutional requirements. Written informed consent for participation was not required for this study in accordance with the national legislation and the institutional requirements.

## Author Contributions

SR was the main author of this study, proposed this idea, developed the questionnaire, and helped in the discussion regarding estimation and write up. WH and MS have equally contributed in discussions, estimations and write-up. All authors contributed to the article and approved the submitted version.

## Conflict of Interest

The authors declare that the research was conducted in the absence of any commercial or financial relationships that could be construed as a potential conflict of interest.

## Footnotes

1 www.sas.com

## References

[B1] Aamir U. B. Badar N. Mehmood M. R. Nisar N. Suleman R. M. Shaukat S. (2012). Molecular epidemiology of influenza A (H1N1) pdm09 viruses from pakistan in 2009–2010. *PLoS One* 7:e41866. 10.1371/journal.pone.0041866 22916112 PMC3423401

[B2] Al-Rabiaah A. Temsah M.-H. Al-Eyadhy A. A. Hasan G. M. Al-Zamil F. Al-Subaie S. (2020). Middle east respiratory syndrome-corona virus (MERS-CoV) associated stress among medical students at a university teaching hospital in saudi arabia. *J. Infec. Public Health* 13 687–691. 10.1016/j.jiph.2020.01.005 32001194 PMC7102651

[B3] Cacioppo J. T. Hawkley L. C. (2009). Perceived social isolation and cognition. *Trends Cogn. Sci.* 13 447–454. 10.1016/j.tics.2009.06.005 19726219 PMC2752489

[B4] Cacioppo J. T. Hawkley L. C. Crawford L. E. Ernst J. M. Burleson M. H. Kowalewski R. B. (2002). Loneliness and health: potential mechanisms. *Psychosomatic Med.* 64 407–417. 10.1097/00006842-200205000-00005 12021415

[B5] Cherif A. Barley K. Hurtado M. (2016). Homo-psychologicus: reactionary behavioural aspects of epidemics. *Epidemics* 14 45–53. 10.1016/j.epidem.2015.09.003 26972513

[B6] DeVellis R. F. (1991). *Scale development: Theory and applications.* Newbury Park, CA: Sage Publications.

[B7] Fardin M. A. (2020). COVID-19 and anxiety: a review of psychological impacts of infectious disease outbreaks. *Arch. Clin. Infect. Dis.* 15:e102779 10.5812/archcid.102779

[B8] González J. M. Regúlez-Castillo M. Vidal-Meliá C. (2016). *A Procedure for Selecting Representative Subsamples of a Population from a Simple Random Sample.* Available Online atSSRN: https://ssrn.com/abstract=2655972. [accessed on Mar 16, 2016].

[B9] Hakim S. T. Tayyab S. M. Qasmi S. U. Nadeem S. G. (2011). An experience with dengue in pakistan: an expanding problem. *Ibnosina J. Med. Biomed. Sci.* 3 3–8. 10.4103/1947-489x.210848

[B10] Holt-Lunstad J. Smith T. B. Layton J. B. (2010). Social relationships and mortality risk: a meta-analytic review. *PLoS Med* 7:e1000316. 10.1371/journal.pmed.1000316 20668659 PMC2910600

[B11] Jowell R. Eva G. (2009). Happiness is not enough: cognitive judgements as indicators of national wellbeing. *Soc. Indic. Res.* 91 317–328. 10.1007/s11205-008-9343-3

[B12] Kaplan J. Frias L. McFall-Johnsen M. (2020). *A Third of the Global Population is on Coronavirus Lockdown – Here’s our Constantly Updated List of Countries and Restrictions.* Available online at: https://www.businessinsider.com/countries-on-lockdown-coronavirus-italy-2020-3

[B13] Khan M. H. Khan H. Sarwar G. Iftikhar B. (2008). Study of obese persons profile at Di Khan, NWFP, Pakistan. *Gomal. J. Med. Sci.* 6 77–80.

[B14] Krejcie R. V. Morgan D. W. (1970). Determining sample size for research activities. *Educat. Psychol. Meas.* 30 607–610. 10.1177/001316447003000308

[B15] Lee S. M. Kang W. S. Cho A. R. Kim T. Park J. K. (2018). Psychological impact of the 2015 MERS outbreak on hospital workers and quarantined hemodialysis patients. *Compr. Psychiatry* 87 123–127. 10.1016/j.comppsych.2018.10.003 30343247 PMC7094631

[B16] Leung G. Lam T. Ho L. Ho S. Chan B. Wong I. (2003). The impact of community psychological responses on outbreak control for severe acute respiratory syndrome in hong kong. *J. Epidemiol. Commun. Health* 57 857–863. 10.1136/jech.57.11.857 14600110 PMC1732323

[B17] Leung G. Quah S. Ho L. Ho S. Hedley A. Lee H. (2009). Community psycho-behavioural surveillance and related impact on outbreak control in hong kong and singapore during the SARS epidemic. *Hong Kong Med. J.* 15 30–34. 20393223

[B18] Levy S. Guttman L. (1975). On the multivariate structure of wellbeing. *Soc. Indic. Res.* 2 361–388. 10.1007/bf00293253

[B19] Levy S. Sabbagh C. (2008). The wellbeing of the self’s personality: a structural analysis. *Soc. Indic. Res.* 89 473–485. 10.1007/s11205-008-9244-5

[B20] Lovibond P. F. Lovibond S. H. (1995). The structure of negative emotional states: comparison of the depression anxiety stress scales (DASS) with the beck depression and anxiety inventories. *Behav. Res. Ther.* 33 335–343. 10.1016/0005-7967(94)00075-u 7726811

[B21] Paek H.-J. Hilyard K. Freimuth V. S. Barge J. K. Mindlin M. (2008). Public support for government actions during a flu pandemic: lessons learned from a statewide survey. *Health Promot. Pract.* 9 60S–72S. 18936261 10.1177/1524839908322114

[B22] Papke L. E. Wooldridge J. M. (1996). Econometric methods for fractional response variables with an application to 401 (k) plan participation rates. *J. Appl. Econom.* 11 619–632. 10.1002/(sici)1099-1255(199611)11:6<619::aid-jae418>3.0.co;2-1

[B23] Pellmar T. C. Brandt E. N. Baird M. A. (2002). Health and behavior: the interplay of biological, behavioral, and social influences: summary of an institute of medicine report. *Am. J. Health. Promot.* 16 206–219. 10.4278/0890-1171-16.4.206 11913326

[B24] Pettersson H. Manley B. Hernandez S. (2020). *Tracking Coronavirus’ Global Spread.* https://www.cnn.com/interactive/2020/health/coronavirus-maps-and-cases [accessed May 29, 2020].

[B25] Rubin G. J. Potts H. Michie S. (2010). The impact of communications about swine flu (influenza A H1N1v) on public responses to the outbreak: results from 36 national telephone surveys in the UK. *Health Technol. Assess.* 14 183–266. 20630124 10.3310/hta14340-03

[B26] Saqib M. Sieberg A. Hussain M. H. Mansoor M. K. Zohaib A. Lattwein E. (2017). Serologic evidence for MERS-CoV infection in dromedary camels. punjab, pakistan, 2012–2015. *Emerg. Infect. Dis.* 23:550. 10.3201/eid2303.161285 28221127 PMC5382745

[B27] Saqlain M. Munir M. M. Ahmed A. Tahir A. H. Kamran S. (2020). Is Pakistan prepared to tackle the coronavirus epidemic? *Drugs Ther. Perspect.* 36 213–214. 10.1007/s40267-020-00721-1 32218652 PMC7095264

[B28] Satici B. Gocet-Tekin E. Deniz M. E. Satici S. A. (2020). Adaptation of the fear of COVID-19 Scale: its association with psychological distress and life satisfaction in turkey. *Int. J. Ment. Health Addict.* 2020 1–9. 10.1007/s11469-020-00294-0 PMC7207987 32395095

[B29] Thissen D. Steinberg L. Kuang D. (2002). Quick and easy implementation of the benjamini-hochberg procedure for controlling the false positive rate in multiple comparisons. *J. Educ. Behav. Stat.* 27 77–83. 10.3102/10769986027001077

[B30] United Nations. (2020). *World Happiness Report.* Available online at: https://happiness-report.s3.amazonaws.com/2020/WHR20.pdf

[B31] Van Bortel T. Basnayake A. Wurie F. Jambai M. Koroma A. S. Muana A. T. (2016). Psychosocial effects of an Ebola outbreak at individual, community and international levels. *Bull. World Health Organ.* 94 210–214. 10.2471/BLT.15.158543 26966332 PMC4773931

[B32] Wang C. Pan R. Wan X. Tan Y. Xu L. Ho C. S. (2020). Immediate psychological responses and associated factors during the initial stage of the 2019 coronavirus disease (COVID-19) epidemic among the general population in china. *Int. J. Environ. Res. Public Health* 17:1729. 10.3390/ijerph17051729 32155789 PMC7084952

[B33] World Health Organization [WHO]. (2020). *Pneumonia of Unknown Cause-China’, Emergencies Preparedness, Response. Disease Outbreak News.* Available online at: https://www.who.int/csr/don/05-january-2020-pneumonia-of-unkown-cause-china/en/

[B34] Wu W. Zheng J. Fang Q. (2020). How a typhoon event transforms public risk perception of climate change: a study in china. *J. Cleaner Prod.* 261:121163 10.1016/j.jclepro.2020.121163

[B35] Xu K. Cai H. Shen Y. Ni Q. Chen Y. Hu S. (2020). Management of corona virus disease-19 (COVID-19): the Zhejiang experience. *J. Zhejiang Univ. (Med. Sci.)* 49 147–157. 10.3785/j.issn.1008-9292.2020.02.02 PMC8800711 32391658

[B36] Zacher H. Rudolph C. W. (2020). Individual differences and changes in subjective wellbeing during the early stages of the COVID-19 pandemic. *Am. Psychol.* 2020:32700938. 10.1037/amp0000702 32700938

